# Energy-Efficient Geopolymer Composites Containing Phase-Change Materials—Comparison of Different Contents and Types

**DOI:** 10.3390/ma17194712

**Published:** 2024-09-25

**Authors:** Agnieszka Przybek, Michał Łach, Rafał Bogucki, Justyna Ciemnicka, Karol Prałat, Artur Koper, Kinga Korniejenko, Adam Masłoń

**Affiliations:** 1 Faculty of Material Engineering and Physics, Cracow University of Technology, Jana Pawła II 37, 31-864 Cracow, Poland; agnieszka.przybek@pk.edu.pl (A.P.); michal.lach@pk.edu.pl (M.Ł.); rafal.bogucki@pk.edu.pl (R.B.); kinga.korniejenko@pk.edu.pl (K.K.); 2 Faculty of Civil Engineering, Mechanics and Petrochemistry, Warsaw University of Technology, Łukasiewicza 17, 09-400 Płock, Poland; justyna.ciemnicka@pw.edu.pl (J.C.); karol.pralat@pw.edu.pl (K.P.); artur.koper@pw.edu.pl (A.K.); 3 Department of Environmental Engineering and Chemistry, Rzeszów University of Technology, Powstańców Warszawy 6, 35-959 Rzeszów, Poland

**Keywords:** geopolymer foams, inorganic polymer composite, phase-change materials (PCM), functionally graded materials and structures for energy saving, composites for modern building insulation materials

## Abstract

The purpose of this study was to analyze the effects of phase-change components on the properties of geopolymer foams. Geopolymer foams are lightweight foamed geopolymers that are characterized by a high degree of porosity. Phase change materials, on the other hand, are compounds that, when added to a material, allow it to absorb, store, and then release large amounts of energy. Three types of PCMs, i.e., MikroCaps, GR42, and PX25, were introduced at 15% by weight. Geopolymer materials were produced based on silica fly ash, and hydrogen peroxide H_2_O_2_ was used to foam the geopolymer structure. The PCM geopolymer composites were cured at 60 °C. The produced materials were tested for physical, chemical, and thermal properties. The tests included oxide and mineral composition analysis of the base material, PCM particle size analysis, apparent density and porosity tests on the foams, water leachability tests, thermal tests (λ, Cv, Cp, α), and structural and textural analysis. The most relevant tests to confirm the performance of the phase-change materials were thermal tests. With the introduction of PCMs, volumetric heat capacity increased by as much as 41% and specific heat by 45%, and thermal diffusivity decreased by 23%. The results confirm the great potential of geopolymer composites as modern insulation materials for buildings and structures.

## 1. Introduction

Numerous attempts and projects are being undertaken around the world to develop low-carbon materials as well as advanced thermal insulation materials [1,2,3]. In many countries, decarbonization policies are being implemented and special emphasis is being placed on solutions aimed at implementing materials with a low carbon footprint, among other things. This issue will become increasingly important in the years to come. In addition, innovative materials with a reduced carbon footprint and better insulation performance are capable of contributing to significant financial savings associated with reduced energy demand [4,5]. The renewable energy industry is developing very intensively, but this development should go hand in hand with the development of modern building materials [6,7]. The introduction of increasingly stringent heat transfer coefficient requirements for building materials and the drive for widespread adoption of passive construction are driving the development of modern building materials with thermal insulation properties [8,9,10]. Current solutions that are used on a large scale are still based on insulation using materials such as polystyrene, polyurethane, or phenolic foams. These materials, in addition to being flammable, are also harmful to the environment and human health and cause significant problems relating to their disposal. Currently, the use of polystyrene foam for insulating buildings is being very strongly restricted (or attempts are being made to restrict it) in Europe and across the world [11]. Comprehensive studies carried out within the scope of REACH regulations by accredited European laboratories have ruled out any adverse environmental impact of products made based on ashes and slag from coal combustion, thus providing a guarantee of the safety of their use, unlike polymer foams. Materials created using UPS are part of a low-carbon economy, as proven by the Tefra Joint Implementation Project in relation to the UN Climate Convention standards, carried out by Ekotech—Ash Engineering. The project showed that each ton of UPS used in place of a portion of cement or lime reduced greenhouse gas emissions by about 0.5 tons [12]. For the production of geopolymers, waste substances from the energy industry (fly ash) and all kinds of deposits from the mining industry are used. The use of post-mining waste is in line with the principles of Zero-Waste Europe, Resource-Efficient Europe, and the circular economy. At present, due to the tightening requirements affecting energy consumption and ecology, it is becoming necessary to look for new alternatives to those currently in use. New solutions should guarantee some improvement in energy efficiency [13,14,15,16,17,18,19,20].

Geopolymer foams are a topic of consideration for many researchers [21,22,23,24,25]. In addition to the synthesis of the foams themselves, the properties of the produced insulations are also key [26,27,28,29,30,31]. The addition of PCMs to geopolymers and geopolymer foams has been the subject of many scientific studies reported in the literature [32,33]. The authors of many papers confirm that the addition of PCMs contributes to improving the accumulation performance of building materials. PCM geopolymers have been an interesting material for construction applications for many years, but they require continuous development and advanced research; possibilities for their use and analysis of the properties have been presented in scientific papers [34,35]. Researchers [33] proved that the addition of a PCM in the form of a composite of paraffin with expanded perlite to geopolymer concrete in amount of 15 wt.% and 30 wt.% reduced the peak temperature in the test room by 1.85 °C and 3.76 °C, respectively, while increasing the heat capacity by 105% and 181%. Despite the reduction in mechanical properties of the geopolymer concrete with PCM, the tested geopolymer concrete containing the PCM showed increased mechanical properties. A very interesting study on the issue of geopolymers with PCMs was presented by the authors of another paper [36], who reported the results of a study related to the addition of innovative fatty acid-based PCMs encapsulated in polyurethane foam to geopolymers. The results of their study indicated that the prepared geopolymer mortar with the developed PU@PCM composite could provide a suitable operating temperature range (10–22 °C) for the design of energy-efficient buildings with good energy storage capacity (ΔHf = 164 J/g). Although the addition of PU@PCM caused a loss of compressive strength, the developed composites still met the mechanical requirements for concrete applications, and the compressive strength of the PU@PCM-rich composite reached 29.5 MPa. In addition, the thermal performance study results showed that the incorporation of PU@PCM significantly improved the heat capacity and slightly reduced the thermal conductivity of the developed composites. A reduction in the mechanical strength of geopolymer composites due to the addition of PCM materials was also demonstrated by the authors of a further paper [35]. The authors of that work showed that micro-encapsulated PCMs (MPCMs), such as E-EVA and St-DVB, slightly reduced the compressive strength of geopolymers, but the composites still showed high strength compared with the typical strength classes of ordinary concrete. It was also proved that the workability of geopolymer concrete remained within acceptable ranges when PCMs were added in small amounts. In addition, a significant increase in heat capacity at the melting point of PCM geopolymer mortars and concretes has been reported, which can be used to save energy in buildings. The effect of PCMs on the mechanical strength of autoclaved concretes was also studied [37]; the study produced a thermal backfill for mining applications based on autoclaved cellular concrete with the addition of phase change materials (AAC-PCM). A gradual decrease in the strength of the composite accumulation backfill with the addition of AAC-PCM was observed as the amount of PCM increased, but the decrease in strength gradually decreased. The maximum reduction in thermal conductivity was 30%, and the specific heat increased by about 31.4%. The authors proved that doping with a small amount of AAC-PCM can significantly improve the thermal capacity of backfill and provides good strength for practical application in mines. The effect of PCMs on reducing mechanical strength was also observed by the author of another paper [38]. The mechanical properties of mixtures with the addition of mPCM decreased as the PCM content increased. The reduction in compressive strength was up to 50% compared with the control sample. All studies conducted so far on geopolymer materials with addition of PCMs have shown that the addition of phase-change materials significantly increases the heat capacity of such composites. Despite the reduction in mechanical properties, it is reasonable to increase the heat capacity and improve the insulating properties. Foamed geopolymer materials with the addition of PCMs deserve special attention here. They have a real chance to replace popular insulating materials such as plastic foams, mineral wools, and polystyrene [39,40]. Tightening environmental requirements may help speed up the implementation of geopolymers as insulation materials on an industrial scale. To reduce CO_2_ emissions by 55% by 2030, it is necessary to use sustainable and energy-efficient materials such as geopolymer concrete or geopolymer foams containing phase-change materials (PCMs) in infrastructure development.

This article presents comprehensive data on foamed geopolymer materials containing phase-change materials. Foamed geopolymers have several favorable characteristics, such as high temperature resistance, resistance to corrosive environments, high heat capacity, and low thermal conductivity. In addition, the use of phase-change materials in the structure of foamed geopolymers contributes to increasing the heat capacity of the entire composite and makes such compositions suitable for use in many scientific fields. The article presents the results of testing foamed composites with the addition of 15 wt.% PCM in three variants. PCM was used both in the form of macrocapsules (granules and powder) and suspension of microcapsules in liquid. An analysis of the physical and chemical properties and very detailed results of the thermal properties of this type of material are presented, as well as an evaluation of its structure. Precise multi-criteria studies, as well as the pursuit of continuous development of these materials, seem inevitable in order to create an opportunity for them to be implemented in the construction industry. The research results presented in the following section of the paper bring new knowledge in the field of properties of geopolymer composites and phase-change materials, and they should have an undeniable impact on the development of disciplines dealing with the topic of sustainable and energy-efficient materials. The authors used an innovative approach, conducting detailed and advanced research.

This article presents an innovative approach to combining low-thermal-conductivity materials with high-heat-capacity materials. Using foamed geopolymers doped with PCMs, it is possible to achieve favorable insulating properties with additional increased heat capacity. The addition of phase-change materials can increase the specific heat of such composites without significantly degrading the insulating properties. This is very attractive from an application point of view, as the use of non-combustible insulation that can accumulate heat can bring huge benefits for mass-scale deployment. Phase-change materials are currently an innovative solution on the market, enabling significant levels of energy savings. Phase-change materials placed in a matrix of porous geopolymer insulation enable heat passing through the building envelope (penetrating) to be additionally retained (captured). After the partition cools down, this heat is given back, but due to the insulating nature of the partition, it does not enter the room in all its quantity but is blocked (insulated) by the porous structure of the matrix material. Such a solution is comprehensive and thermally efficient and guarantees thermal comfort in the rooms where it is used. A building envelope made with the participation of such a solution will not cause overheating of rooms and buildings. The solutions and research results described in this article undoubtedly represent an innovative approach to the use of PCMs in building materials and can contribute to the significant development of such disciplines as civil engineering, environmental engineering, materials engineering, and related fields.

## 2. Materials and Methods

### 2.1. Base Materials

The base material of the geopolymer foams produced was concrete fly ash from the Skawina Heat and Power Plant (CEZ Skawina S.A, Skawina, Poland), certificate no: 1488-CPR-0166/W. According to the data from the document, the density of the fly ash grains was 2210 kg/m^3^, the loss on roasting was in categories A and B, and the fineness of this material was classified in category N. This material had a high content of silicon dioxide (SiO_2_)—about 50–60 wt.% and more than half that content of diglin trioxide (Al_2_O_3_). This fly ash can also be called silica fly ash due to its high SiO_2_ content. Siliceous fly ash is a fine-grained material with pozzolanic properties. It is obtained by mechanically or electrostatically precipitating ash from the waste gases of coal dust combustion in power boilers. Oxide analysis of fly ash was carried out by XRF fluorescence analysis, which was performed on a SCHIMADZU EDX-7200 (SHIMADZU Europa GmbH, Duisburg, Germany). The test was carried out in an air atmosphere with holders designed for bulk materials and Mylar film; the results are shown in Table 1. Mineral phase analysis was carried out by XRD X-ray diffraction analysis on a PANalytical AERIS series instrument (Malvern PANalytical, Lelyweg 1, Almelo, The Netherlands) and is shown in Table 2. Quantitative analysis was carried out using the Rietveld method, which was implemented in HighScore Plus software (version 4.8). The PDF-4+ database of the International Center for Diffraction Data (ICDD) was used during the analysis. Measurements were recorded in the range of 10–100° with a step of 0.003° (2θ) and a step time of 340 s, using Cu Kα radiation.

### 2.2. Phase-Change Materials

Three different organic paraffinic phase-change materials were added to the geopolymer foams. It was decided to compare three types of phase-change materials with different phase-change temperatures ranging from 22 °C to 42 °C. Additionally, these materials differed in the form in which they appeared. The first was MikroCaps PCM 28 Slurry (MikroCaps, Ljubljana, Slovenia), and phase-change materials from Rubitherm (Rubitherm, Berlin, Germany)—GR42 and PX25—were also used. MikroCaps is a material in which microcapsules can be placed in suspension, while the other two materials are macro capsules inside which a paraffinic phase-change material can be placed. Table 3 shows the specifications of all the phase-change additives used.

Using an Anton Paar PSA 1190LD laser analyzer (Anton Paar GmbH, Graz, Austria), particle size analysis was performed on all PCMs used in the geopolymer foams. Wet analysis was performed for the MikroCaps additive (due to the nature of the material), while laser dry analysis was performed for the other components. Five test measurements were taken for each material, and then the mean and standard deviation were calculated using Kalliope Professional software (version 2.22.1). Particle size analysis was performed to see whether the particle size of PCMs affected the properties studied in the next section of this article. Table 4 shows the results of the measurements.

### 2.3. Preparations of Geopolymer Foams

The hydraulic additive stabilizing the porous structure of the produced geopolymer foams was Portland cement with the trade name Górkal 70 (Górka Cement, Trzebinia, Poland). Syringaldehyde (Merck, Darmstadt, Germany) was used as a surfactant. Sand from the Świętochłowice Sand Plant (Świętochłowice, Poland) and ash microspheres, responsible for the formation of closed pores (TERMO-REX S.A., Jaworzno, Poland), were also used in the mixture. Sand and microspheres together constituted the filler in the geopolymer structure. The polycondensation reaction of the geopolymer was induced by adding a 10-mole alkaline solution of sodium silicate with sodium water glass. Sodium silicate R-145 with a molar modulus of 2.5 and a density of 1.45 g/cm^3^ (ANSER Chemical Plant, Wiskitki, Poland) and flaked caustic soda (PCC Group, Brzeg Dolny, Poland) were used. The most important material for making geopolymer foams was 36% hydrogen peroxide H_2_O_2_ (Azoty Group, Puławy, Poland), which was responsible for the formation of the porous geopolymer structure. To the geopolymer foams, 15 wt.% phase-change materials were added, as described above. Since the addition of PCMs was intended to improve both insulation and accumulation properties, it was decided to conduct tests with the addition of the highest acceptable level of PCM. The materials selected for testing contained between 30 wt.% and 60 wt.% PCM in their composition. Their addition in the form of materials intended for the construction industry in the amount of 15 wt.% resulted in an active material capable of phase transformation. This was also observed in the samples from 5% by weight to 10% by weight. Addition of smaller amounts would have little effect and such action would not be effective. On the other hand, the addition of larger amounts of PCM would result in a very high amount of organic matter in the final product, and cost could be a barrier to implementation. Table 5 shows the determinations of the samples produced and the amounts by weight of components used in their production.

**Figure 1:**
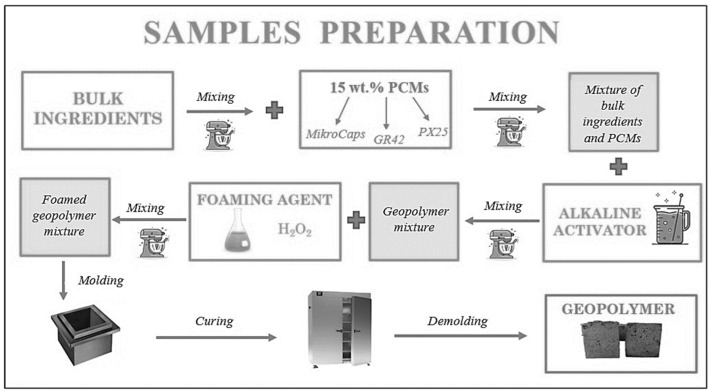
Scheme for manufacturing geopolymer foams with PCM addition.

**Figure 2:**
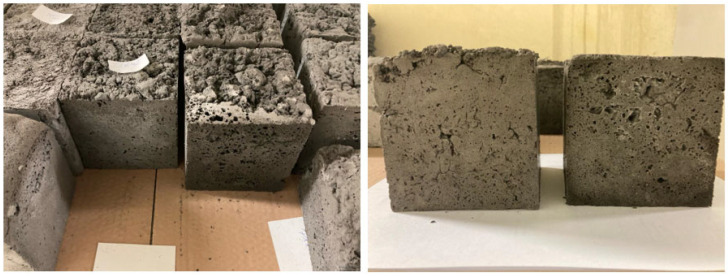
Prepared test samples with PCM additive (overview photo) (samples measuring 10 × 10 × 10 cm).

Fly ash, cement, sand, ash microspheres, and syringaldehyde surfactant were mixed dry in an M/LMB-s laboratory mixer (GEOLAB, Warsaw, Poland) for about 5 min at 58 rpm until all ingredients were evenly mixed. Phase-change additives GR42 and PX25 were mixed with the solid ingredients, and MikroCaps were added after mixing the solid ingredients. After mixing the dry geopolymer mixture and additives, an alkaline activator in the form of a 10 M sodium silicate solution with sodium water glass was introduced and mixed for another 10 min. When a dense mass had been obtained, 36% hydrogen peroxide H_2_O_2_ was added. Once a homogeneous mass was obtained and the formation of a porous structure had been initiated, the material was very quickly transferred to suitable molds (to prevent the foams from sagging) and then annealed at 60 °C for 24 h in an SLW 750 laboratory dryer (POL-EKO Perfect-Environment, Wodzisław Śląski, Poland). After 24 h, the samples were unmolded. The samples for further testing were cubes with dimensions of 10 × 10 × 10 cm. A schematic of the manufacture of geopolymer foams with the addition of phase-change materials is shown in Figure 1, while Figure 2 shows the finished test samples (overview photo).

### 2.4. Tests of Physical Properties—Apparent Density of Ready-Made Samples

The apparent density of the finished cubes was forfeited using the geometric method, based on the mass and volume of the samples—p_b1_. The volume of the slabs was constant (10 × 10 × 10 cm), but the mass varied slightly from sample to sample. The dimensions of the samples were measured using a laboratory caliper to the nearest 0.01 mm, and the weights of the samples were determined to the nearest 0.01 g using a RADWAG PS 200/2000.R2 precision laboratory analytical balance (RADWAG, Radom, Poland).

Knowing the dimensions and mass of the samples, their volume apparent density ρb1 was calculated from the simple relation (1):

(1) $$\rho_{b1}=\frac{m}{V}\;[\frac{kg}{m^{3}}]$$

where m is mass and V is volume.

### 2.5. Tests of Physical Properties—Porosity of Ready Made Sample

Porosity tests were performed on a GE Phoenix v|tomex|m (General Electric Company, Hürth, Germany) with a microfocus lamp using a cone beam. Each sample was scanned with the same scanning parameters—120 kV at a lamp intensity of 350 µA. A copper filter with a constant thickness of 1 mm was used for the tests. The measurement was carried out with an accuracy at which the dimension of the voxel was equivalent to 30 µm. A calibration procedure was carried out according to the manufacturer’s recommendations. A single study involved taking 2.500 images. A single examination lasted about 60 min. During the examination, the module was activated to exclude the influence of a defect in a single detector pixel, as well as an auto-sco function to optimize the geometry. For safety reasons, radiation levels were monitored during and after the study.

### 2.6. Tests of Chemical Properties—Water Leachability of Ready-Made Samples

The next tests that were performed for the prepared samples were leachability tests, which were commissioned by AP Geotechnika (Siemianowice Śląskie, Poland). The AP Geotechnika laboratory provides services in testing the physical and chemical characteristics of aggregates including anthropogenic industrial waste, soils, soil–soil mixtures, and construction materials. For testing, 500 g of powder of each sample was prepared (the samples were crushed using a ball drum mill—model C20-ABLA-1/manufacturer ATEST, Kielce, Poland), which was then tested.

### 2.7. Tests of Mechanical Properties—Compressive Strength

Strength tests were performed using an MTS Criterion 43 machine equipped with TestSuites 1.0 software, with a measuring capacity of up to 30 kN. The test focused on measuring compressive strength, confirming the mechanical integrity of the specimens. The test speed was set at 10 mm/min. In construction, the procedure for determining the compressive strength of concrete samples is outlined in the standard PN-EN 12390-3:2019-07 (Testing of concrete—Part 3: Compressive strength of test specimens). During compression testing, specimens are loaded until they reach a critical point that leads to failure. The maximum applied load is used to calculate the compressive strength of the concrete using a specific formula:

$$R_{c}=\frac{F}{A_{c}}[MPa]$$

where R_c_ is compressive strength [MPa], A_c_ is the cross-sectional area of the specimen on which the compressive force acts [mm^2^], and F is the maximum load.

The compressive strength is reported to the nearest 0.1 MPa, and the type of failure observed helps determine the accuracy of the test.

### 2.8. Tests of Thermal Properties—Thermal Conductivity λ, Volumetric Heat Capacity Cv, Specific Heat Cp, and Thermal Diffusivity α

The thermal tests were carried out at the Department of Civil Engineering, Mechanics and Petrochemistry, Warsaw University of Technology in Plock, Poland. Six measurements were made for each sample using an Isomet 2114 (Applied Precision Ltd., Bratislava, Slovakia) (ASTM standard D5334-08), a commercial microprocessor-controlled device with interchangeable probes. A known heat source produced a radially propagating wave in the sample. Power dissipation generated heat flow through the probes in direct contact with the material, and a serial port (RS-232C protocol) recorded the signal. Semiconductor sensors at specific points on the material sampled the change in temperature as a function of time: the temperature increased linearly with the logarithm of time. The device has a wide measurement range and can be used for insulation and construction materials, among other things. The measurement range depends on the probe used and includes λ values from 0.015 to 6.0 W/(m × K) and Cv values from 0.04 to 3 MJ/(m^3^ × K). The measurement accuracy for the above ranges of thermal conductivity and specific heat by volume was 5%. The meter can employ two optional types of probes: needle probes for soft materials and surface probes for hard materials. Measurement data can be stored in the device’s internal memory or exported to a computer. In the experiment presented here, a surface probe was used. The device analyzed the temperature changes that resulted from the response of the material under testing to the flow of thermal pulses. These changes were measured by interchangeable probes connected to a meter, in turn connected to a computer that recorded the results. During the measurement, the amount of heat generated by the device was known, and the heat propagated radially through the sample. The temperature rise in the sample varied linearly with the logarithm of time. This relationship made it possible to directly obtain the thermal conductivity of the material under testing.

According to the second law of thermodynamics, the thermal conductivity (λ) was determined by Equation (2):

(2) $$\lambda =\frac{Qd}{A∆T}[\frac{W}{m\times K}]$$

where Q is the amount of heat transferred, d is the distance between two isotherms, A is the area, and ∆T is the temperature gradient.

Volumetric heat capacity Cv is the ability of a material to accumulate heat, expressed in terms of the amount of heat needed to heat 1 m^3^ of material by 1 K. The value of Cv was calculated according to Formula (3):

(3) $$C_{v}=\frac{Q}{V_{c}∆T}=C_{p}*p_{b1}[\frac{kJ}{m^{3}\times K}]$$

where Q is the amount of heat transferred, Vc is the volume, ∆T is the temperature gradient, Cp is the specific heat, and p_b1_ is the apparent density.

Specific heat capacity (Cp)/specific heat is the heat required to increase the temperature of 1 g of a substance by 1 K and is given by (4):

(4) $$C_{p}=\frac{Q}{m∆T}=[\frac{J}{kg\times K}]$$

where Q is the amount of heat transferred, m is mass, and ∆T is the temperature gradient.

Thermal diffusivity (α) quantifies the rate of heat transfer of a material from the hot side to the cold side. Here, it was calculated using Equation (5):

(5) $$\alpha =\frac{\lambda }{p_{b1}C_{p}}=[\frac{{mm}^{2}}{sec}]$$

where λ is thermal conductivity, p_b1_ is the apparent density, and Cp isspecific heat.

Thermal measurements were performed on all modified samples, measuring thermal conductivity λ, volumetric heat capacity Cv, and thermal diffusivity a, always performed in series of six measurements. The specific heat Cp, expressed in units of J/(kg × K), was obtained by dividing the measured volumetric heat capacity Cv by the volumetric apparent density of the material p_b1_.

When the standard deviation of a random variable X is unknown, the distribution of the sample mean $\bar{X}$ is very well approximated by Student’s t-distribution. If the random variable under study has an N(µ, σ) distribution and the standard deviation is not known, we can build a confidence interval using Student’s t-distribution with an apparent density probability expressed by Formula (6), where Γ(x) is the Euler gamma function:

(6) $$f\left(t,n\right)=\frac{\Gamma \left(\frac{n+1}{2}\right)}{\Gamma \left(\frac{n}{2}\right)\sqrt{n\pi }}{\left(1+\frac{t^{2}}{n}\right)}^{-\frac{n+1}{2}}$$

After transformations, we finally obtain (7):

(7) $$P\left(\bar{X}{-t}_{n-1;\;\alpha /2}\frac{s}{\sqrt{n}}\leq \mu \leq \bar{X}+t_{n-1;\;\alpha /2}\frac{s}{\sqrt{n}}\right)=1-\alpha$$

where α is the assumed significance level and 1 − α is the confidence level.

### 2.9. Visual Porosity Assessment of Ready Made Samples with PCMs

Image analysis of the fabricated PCM geopolymer foams was performed on a Keyence VHX-7000 digital optical microscope (KEYENCE INTERNATIONAL, Mechelen, Belgium). Photographs of the porous structures of the material were taken using the 3D function.

### 2.10. Microstructure of Ready Made-Samples

A JEOL IT200 SEM scanning microscope (JEOL, Akishima, Tokyo, Japan) was used to take images of the microstructure of the finished geopolymer foams. Samples for SEM studies were properly prepared in advance. The small sections of the samples taken were cleaned of dust and particles formed during the separation process and were then dried to a constant mass at 40 °C (so as not to lead to changes in the structure of the PCMs). To perform the observations on the SEM microscope, the materials were attached to special carbon disks and placed on metal tables and then in a holder. A special EM-Tec C33 carbon adhesive was also used to better attach the material, leading to better conduction. The surface of the material was coated with a conductive gold layer using a DII-29030SCTR Smart Coater vacuum sputtering machine (JEOL Ltd., Peabody, MA, USA).

### 2.11. Sample Conditioning Tests under Varying Humidity Conditions

Conditioning tests were conducted on 200 × 200 × 2.5 mm samples. Two tests were conducted. The first consisted of immersing the sample in water at 50% of its volume at room temperature. The second test consisted of leaving the sample in winter conditions for 2 weeks. Humidity in room conditions was 40–60%, while in winter conditions, it was about 20%.

## 3. Results

### 3.1. Tests of Physical Properties—Apparent Density of Ready-Made Samples

As described above, four types of samples were made: a reference sample containing no PCM materials, and three samples with 15 wt.% phase-change material. Each variant was performed four times. Each sample tested had dimensions of 100 × 100 × 100 mm. The results presented below are given for all four samples from each variant due to the significant scatter in the results. Such discrepancies are common for foamed geopolymer samples. Each of the described samples was tested six times and the average results of the six measurements taken are presented.

The average apparent density values ρb1 of the tested geopolymers, are summarized in Table 6. Based on eight measurement results for each variant of the samples, the average value was also calculated to better illustrate the relationship between the content of phase-change materials and the apparent density.

From the above measurement data, it can be seen that each phase-change additive caused a decrease in apparent density. The 15 wt.% MikroCaps caused a 6% decrease in apparent density, and this was the largest change. The 15 wt.% GR42 additive decreased the apparent density by 4%, and 15 wt.% PX25 decreased the apparent density by only 1%. The decrease in apparent density in all cases was not large, but it was significant since the phase-change additives increased the apparent density of the samples tested. The decrease in the apparent density of the obtained mixtures compared with the reference sample was most likely to have been dictated by a change in their consistency. It was also related to a change in the surface tension of the mixture, by which the formation of the porous structure proceeded differently. This was particularly evident in the case of the sample with the addition of MikroCaps, which was added in liquid form. Introducing 15% of this additive resulted in a lower apparent density than in samples without the additive. The introduced PCMs affected the consistency of the geopolymer mixtures and the stability of the produced foams. However, confirmation of this influence/phenomenon requires further comprehensive studies.

### 3.2. Tests of Physical Properties—Porosity of Ready-Made Samples

**Figure 3:**
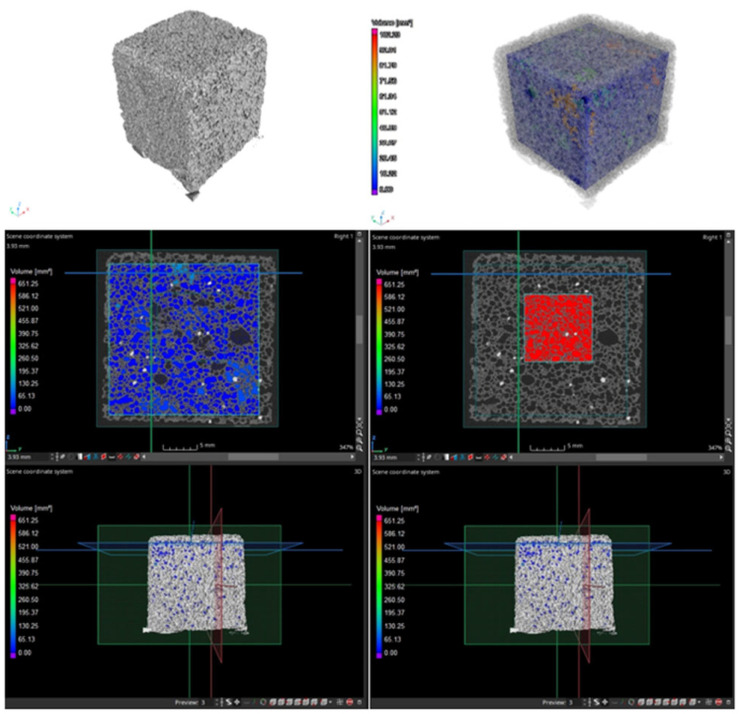
Scans from the CT scanner for the 15 wt.% MikroCaps sample and the areas used to calculate sample porosity (blue and red colors).

**Figure 4:**
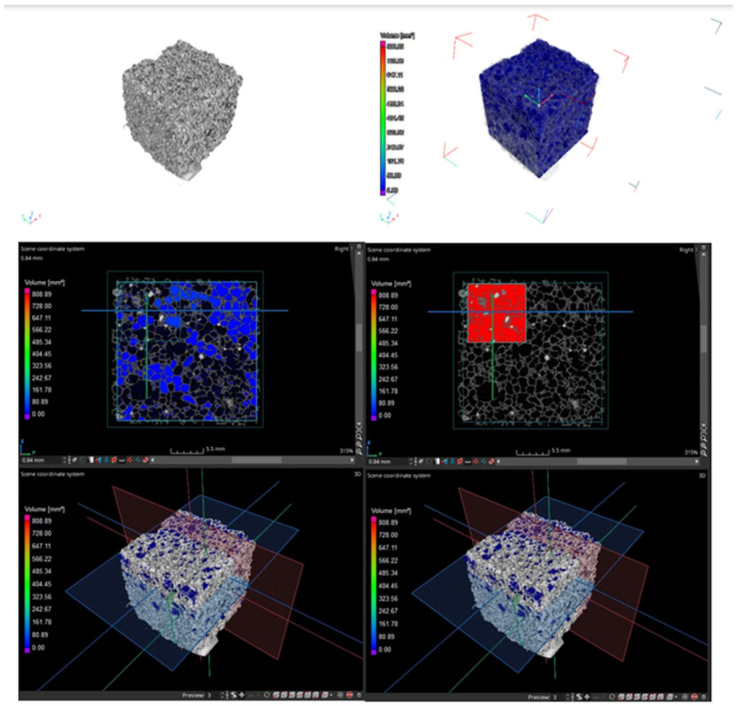
Scans from the CT scanner for the 15 wt.% GR42 sample and the areas used to calculate the porosity of the samples (blue and red colors).

Since this research should be considered only complementary, only two (25 × 25 × 25 mm) samples were tested (due to the high cost of the testing). It was decided to conduct tests only for samples containing PCMs in two variants: PCM in the form of MikroCaps suspension and one variant of PCM in the form of macrocapsules—GR42. The porosity of the samples with 15 wt.% MikroCaps and 15 wt.% GR42 was checked, and the results are shown in Table 7. Figure 3 and Figure 4 show scans of the samples taken with the CT scanner described above. The large regions are shown in blue, while the small closed regions are shown in red. Both of these regions were used to calculate the porosity, as shown in the table below.

The higher porosity in the large region was from the sample with 15 wt.% MikroCaps—51%, while in the small closed region, the higher porosity was from the sample with 15 wt.% GR42—81%. The more authoritative result was the one from the small closed region; so, the higher porosity was obtained from the sample with 15 wt.% GR42, as confirmed by the above scans.

### 3.3. Tests of Chemical Properties—Water Leachability of Ready Made Sample

Table 8 shows the results of testing the concentrations of harmful substances, along with the limit values. In addition, the pH of the tested samples and chromium (VI) content were also determined. Metals, chlorides, fluorides, sulfates, heavy metals, and chromium (VI) in the leachate were tested according to ISO 17,025 by the laboratory’s internal methods. Moisture content, dissolved solids (TDSs), and dissolved organic carbon in the leachate were tested by non-accredited in-house methods. All harmful substances described so far were determined wet, while pH in the leachate was determined for a dry sample.

The above table shows the permissible leaching limits. Fly ash, which is the base material of the samples made, is categorized as inert waste. When alkaline solutions are added to the geopolymer mixture, the produced product should be treated as non-hazardous and inert waste. In relation to the criteria for allowing hazardous waste to be disposed of in hazardous waste landfills, all tested samples had lower permissible values of all elements listed in the table (for hazardous waste). Regarding the pH of the tested samples, each of them showed an alkaline pH, due to activation of the polycondensation process with an alkaline solution of NaOH. In Poland, all waste must be stored in accordance with the storage criteria described in the table. This test was conducted to confirm that the materials produced do not pose a threat to the environment. Exceeding leachability levels for materials classified as inert may be the result of the use of alkaline activators, in the presence of which heavy metals and other elements leach from the fly ash. Industrial application of the solution may require additional special treatment involving leaching (flushing) of the final product to get rid of undesirable leachable components [43].

### 3.4. Mechanical Properties Testing—Compressive Strength

The compressive strength R_c_ was determined as the average value from 10 measurements. Specimens with dimensions of 40 × 40 × 40 mm were prepared for this experiment. Table 9 shows the results of compressive strength testing for all samples.

Compressive strength tests were conducted to determine the mechanical properties of the geopolymer composites. The tests showed that the MikroCaps phase-change additive did not affect the compressive strength. The GR42 additive increased the compressive strength by 30%, while PX25 decreased this parameter by 43%. GR42 proved to be the best component due to its material characteristics, containing hard, brown granules that improved the strength properties.

### 3.5. Tests of Thermal Properties—Thermal Conductivity λ, Volumetric Heat Capacity Cv, Specific Heat Cp, and Thermal Diffusivity α

The measurement data above represent four measurements for each given sample variant (R.F.A, 15 wt.% MikroCaps, 15 wt.% GR42, 15 wt.% PX25). The dimensions for each test sample were 100 × 100 × 100 mm. Based on the average values $\left(\bar{X\;}\right)$ from the table, the average value was calculated for the four sample variants. These results are shown in Table 10 to better illustrate the percentage differences between the composites.

With the results of n measurements, parameters such as the mean $\bar{X}$ and standard deviation calculated from the sample were determined. The ranges in which the actual measured values of thermal conductivity λ, specific heat Cp, and thermal diffusivity were located were estimated according to a specified probability. An assessment of measurement uncertainty based on Student’s t-distribution was used for all measurements carried out. Based on statistical calculations, with an assumed confidence level of 95%, the confidence intervals of the measured thermal properties were determined. With a probability close to unity, the sought-after values of the thermal parameters (λ, Cp, α) of the modified geopolymers were within the ranges shown in Table 11.

According to analysis of the data in Table 10, the thermal conductivity of all samples oscillated at the same level of 0.08 W/m·K. The thermal conductivity for the 15 wt.% MikroCaps sample decreased by 0.1% and for the 15 wt.% GR42 sample, it decreased by 4%, while for the 15 wt.% PX25 sample, it increased by 9%. All thermal conductivity values were very low, even though that the thickness of these samples was as much as 10 cm. The volumetric heat capacity for each sample with PCM increased significantly, a very welcome effect. The largest increase was observed for the sample with 15 wt.% MikroCaps—a 41% increase. The Cv for the sample with 15 wt.% GR42 increased by 13%, and that for the sample with 15 wt.% PX25 also increased by 41%. Regarding the specific heat, all samples with PCMs again had higher Cp values than the reference sample. The Cp of the sample with 15 wt.% MikroCaps increased by 45%, that of the sample with 15 wt.% GR42 increased by 19%, and that of the sample with 15 wt.% PX25 increased by 44%. The last parameter analyzed was the thermal diffusivity coefficient a. The coefficient a indicates the rate at which temperature changes from one plane to another, i.e., the susceptibility of the material to temperature equalization during heating or cooling at specific locations. The a coefficient should be as low as possible because then, there is greater susceptibility of the material to temperature equalization. All samples with PCMs had a lower a than the reference sample. For the sample with 15 wt.% MikroCaps, the thermal diffusivity decreased by 23%, for the sample with 15 wt.% GR42 by 14%, and for the sample with 15 wt.% PX25, it decreased by 20%. All parameters that were expected to improve with the addition of PCMs demonstrated very favorable results, so all tests confirmed the effective performance of the phase-change materials in the geopolymer foams. All samples obtained the same confidence level—95%. This means that 95% of the measurement results were within the real range. The most favorable results considering all three studied parameters were obtained for mixtures modified with the addition of MikroCaps. On the one hand, this can be attributed to the lowest apparent apparent density of these materials, but most importantly it is related to the highest heat capacity [kJ/kg]. This value was more than three times higher for MikroCaps than for GR42, for example (Table 3).

### 3.6. Visual Porosity Assessment of Ready-Made Samples with PCMs

**Figure 5:**
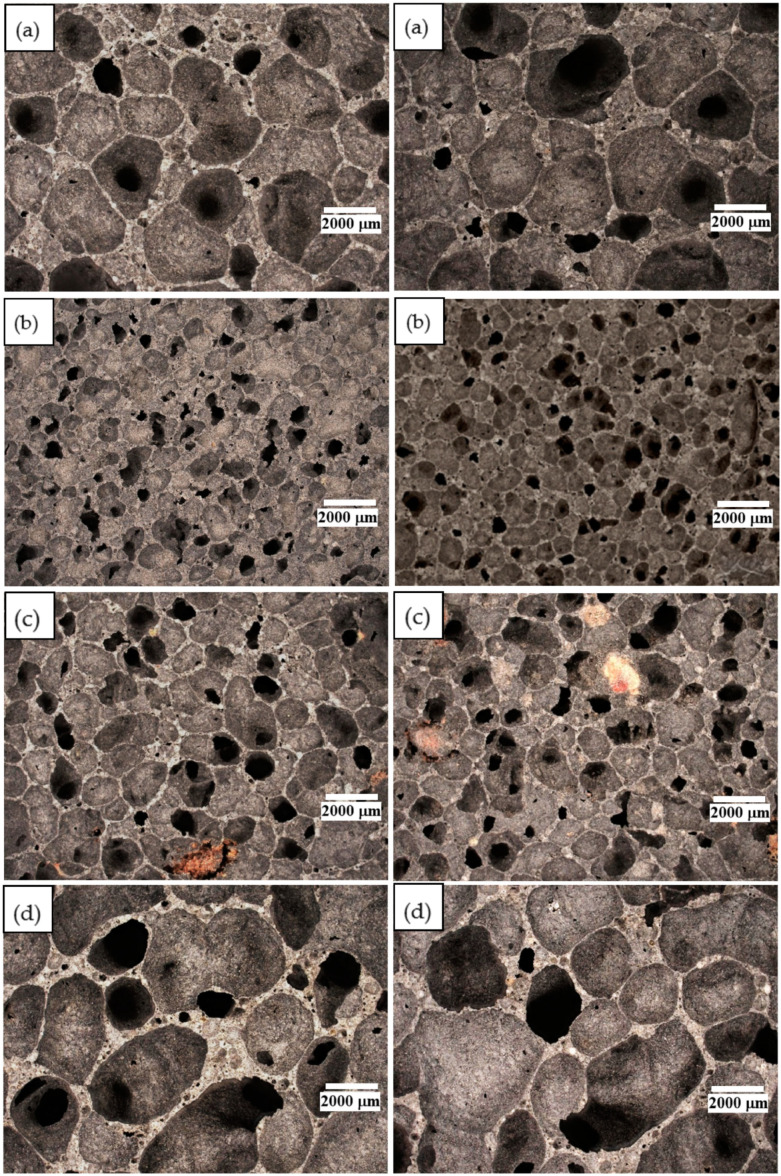
Porous structure morphology: (**a**) reference fly ash; (**b**) 15% MikroCaps; (**c**) 15% GR42; (**d**) 15% PX25.

The results of this test are shown in Figure 5. A magnification of 20× was used for each sample. The marker in the figures corresponds to 2000 μm.

Analysis of the structure by digital optical microscopy made it possible to determine the size of the pores and their qualitative contribution. All images show the macropores of the structures produced. These were mainly closed pores. It was observed that the reference ash sample was characterized by medium-sized pores with high heterogeneity. Pores of 1000–3000 μm were observed in the reference sample. The sample with 15 wt.% MikroCaps had very fine and homogeneous pores of 500–1000 μm. The pores in the sample with GR42 additive were slightly larger than in the sample with 15 wt.% MikroCaps and were quite heterogeneous, with a size of 750–1500 μm. The addition of PX25 made the pores homogeneous and similar in size, around 1500–3500 μm, with an average size of 2000 μm. Based on the results of the study, it can be concluded that the particle size of the phase-change materials used had a significant effect on porosity. The average particle size of the MikroCaps additive resulted in the formation of fine and homogeneous pores. With a slightly larger particle size than MikroCaps, PX25 obtained medium-sized homogeneous pores. The large particle size of the GR42 additive made the pores quite heterogeneous and of different sizes.

### 3.7. Microstructure of Ready-Made Samples

**Figure 6:**
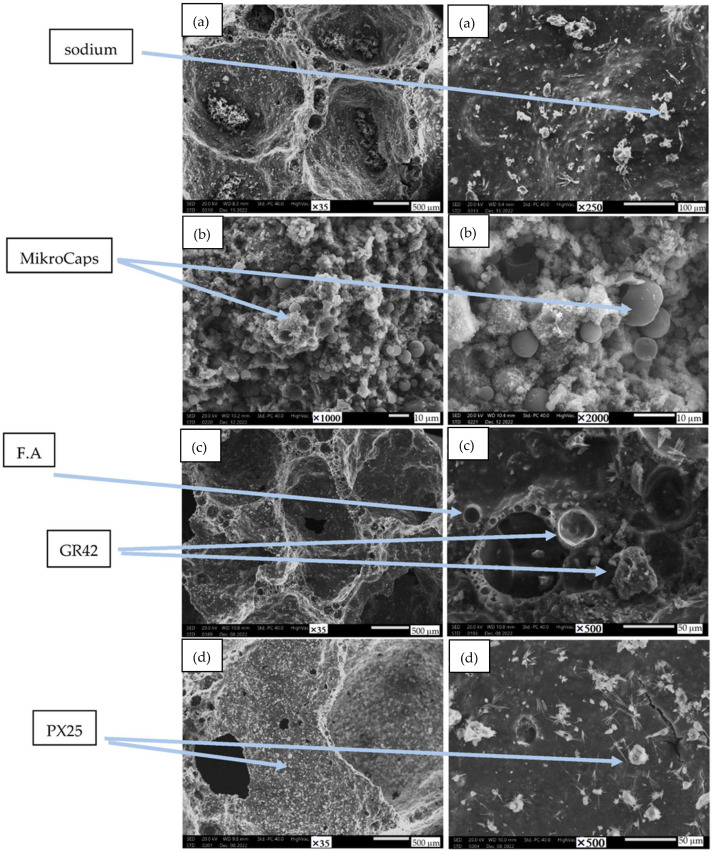
Microstructure of the sample: (**a**) reference fly ash, (**b**) 15% MikroCaps, (**c**) 15% GR42, (**d**) 15% PX25.

Figure 6 shows the morphology of geopolymer structures with the addition of phase-change materials. The photos were taken at various magnifications to illustrate the porosity and phase-change additives contained in the samples.

The SEM image above shows the porous and amorphous structure of pure fly ash-based geopolymer and samples with phase-change materials added. The arrows indicate undissolved ash particles and show unbound sodium particles and paraffin PCM additives, which are visible at high magnification. Paraffin binds very well to the geopolymer matrix, as evidenced by the above illustrations. The SEM images also confirm the effects achieved with PCM additives related to insulation and accumulation parameters. MikroCaps particles are most visible on these microphotographs, as they are present in large numbers and are evenly distributed. For other materials such as GR42 and PX25, as a result of the fact that they have larger particles, the numbers of these particles are less apparent and they are more difficult to identify in the images. MikroCaps occurring in large quantities throughout the material gave the best results related to thermal storage parameters and this assessment was confirmed by the SEM images.

### 3.8. Sample Conditioning Tests under Varying Humidity Conditions

**Figure 7:**
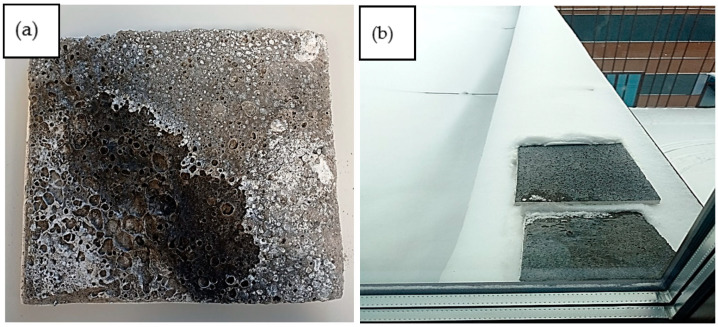
Conditioning tests under varying humidity conditions: (**a**) room temperature; (**b**) winter conditions.

Figure 7 shows the results of this testing; sodium efflorescence appeared on the samples after immersion in water and conditioning at room temperature. This natural phenomenon was related to the fact that the precursor of the geopolymerization reaction was 10 M NaOH. Under winter conditions, no efflorescence appeared on the samples. The addition of PCMs did not affect the formation of efflorescence.

## 4. Discussion

In this article, a series of comprehensive studies analyzing samples of 15 wt.% phase-change material were conducted. This article presents studies of the physical properties and chemical properties, extensive studies of the thermal properties (to check the real performance of these composites), and complementary qualitative studies of the structures. In the discussion section, the authors analyze the most relevant relationships.

From the above measurement data, it can be seen that each phase-change additive caused a decrease in apparent density. The 15 wt.% MikroCaps caused a 6% decrease in apparent density, and this was the largest change. The 15 wt.% GR42 additive decreased the apparent density by 4%, and 15 wt.% PX25 decreased the apparent density by only 1%. The decrease in apparent density in all cases was not large, but it was significant since phase-change additives in such content should increase the apparent density of the samples tested. The decrease in apparent density may have been caused by inaccurate mixing of the ingredients during the manufacture of the samples or failure of all the ingredients to react. The decrease may also have been because the consistency of the mixture changed due to the addition of the phase-change materials, and despite the addition of a higher apparent density material to the foamed geopolymer mixture, a structure with a higher degree of foaming was obtained. In the work of other authors, it was observed that the decrease in apparent density was closely related to the porosity of the geopolymer structure and the size of the pores. As the porosity increased, a decrease in the roundness of the voids was observed [44,45]. In this work, the porosity of two samples of 15 wt.% MikroCaps and 15 wt.% GR42 was studied. The sample with 15 wt.% GR42 had a higher porosity of 81%. The high porosity of this sample caused the pores to be much less rounded and homogeneous than those of the MikroCaps sample (the porosity of this sample was lower, and the pores were more homogeneous and had similar shapes and sizes). The formation of increasingly fine voids was mainly responsible for the reduced apparent density in these samples [46,47]. Previous findings are confirmed by the present work. The largest decrease in apparent density was observed for the MikroCaps sample, which was characterized by very fine pores. For the sample with 15 wt.% GR42, the pores were slightly larger than those of the MikroCaps sample, resulting in a smaller decrease in apparent density. The smallest decrease in apparent density was observed for the sample with PX25—only 1%. The pores of this sample were quite large and there were far fewer of them than in the previous two cases. Porosity is also related to the size of the introduced additive. Individual microcapsules of small size (3–10 μm) can fill the voids between aggregates, leading to a reduction in porosity [48,49]. The porosity of the sample with MikroCaps was lower than that of GR42, and the particle size of this additive was small and within the range stated above (6 μm), making the porosity decrease compared with GR42.

Analysis of the strength properties showed that the MikroCaps phase-change additive did not affect the compressive strength. The GR42 additive increased the compressive strength by 30%, while PX25 decreased this parameter by 43%. GR42 proved to be the best component due to its material characteristics; its hard, brown granules improved its strength properties. The suspension of microcapsules in water did not affect the strength characteristics of geopolymer foams at 15 wt.%. GR42, consisting of large brown granules, enhanced the strength of the geopolymer foams. However, the addition of a modifier, presented as a fine white powder made up of small microcapsules, led to a reduction in strength properties. Researchers in the USA [50] examined the impact of PCMs on strength properties and found that while PCM addition slightly lowered the compressive strength of geopolymer mortar, the decrease was minimal. Even with up to 20% PCM, the compressive strength remained sufficient for construction purposes.

The thermal conductivity of all samples oscillated at the same level of 0.08 W/m·K. The thermal conductivity of the 15 wt.% MikroCaps sample decreased by 0.1%, that of the 15 wt.% GR42 sample decreased by 4%, while that of the 15 wt.% PX25 sample increased by 9%. All thermal conductivity values were very low, even though the thickness of the samples was as much as 10 cm. The reduction in conductivity is an indirect effect since the addition of PCM alone does not reduce the thermal conductivity coefficient. The increase in thermal conductivity is determined by a reduction in the porosity of the insulating material [51,52]. Thermal conductivity for similar unconventional natural raw materials ranges from 0.06 to 0.1 W/m·K [53,54], which is in close agreement with measured values. The largest increase in conductivity was observed for the PX25 sample, which had the largest pores, making its porosity the lowest, as shown in the digital optical microscope images. The volumetric heat capacity for each PCM sample increased significantly, representing a very desirable effect. The largest increase was observed for the sample with 15 wt.% MikroCaps—a 41% increase. The Cv for the sample with 15 wt.% GR42 increased by 13%, and for the sample with 15 wt.% PX25, it increased by 41%. Authors of other articles have also shown that the addition of PCMs in large quantities increases heat capacity [55,56]. Regarding specific heat, all samples with PCMs again had higher Cp values than the reference sample. The Cp of the sample with 15 wt.% MikroCaps increased by 45%, that of the sample with 15 wt.% GR42 increased by 19%, and that of the sample with 15 wt.% PX25 increased by 44%. The authors of previous papers [57,58] observed that the higher the PCM content, the better were the specific heat results. The PCM-containing geopolymers showed lower thermal conductivity, slower heating and cooling, and maintained room temperature while reducing temperature fluctuations. In this paper, the addition of PCMs also increased the specific heat. The last parameter analyzed was the thermal diffusivity coefficient a. All samples with PCMs had a lower coefficient a than the reference sample. For the sample with 15 wt.% MikroCaps, the thermal diffusivity decreased by 23%, for the sample with 15 wt.% GR42 by 14%, and for the sample with 15 wt.% PX25, it decreased by 20%. Other researchers have also shown that the thermal diffusivity of PCMs is low [59,60]. All parameters that should improve with the addition of PCMs had very favorable results, so all tests confirmed the significance of the phase-change materials in geopolymer foams. All samples obtained the same confidence level—95%. This means that 95% of the measurement results belonged to the real range.

## 5. Conclusions

Based on the above discussion of the research results, several conclusions can be drawn to summarize this research work:

- The reference ash sample had the highest apparent density among all the geopolymer foams—423 kg/m^3^. Each phase-change additive caused a decrease in apparent density. The 15 wt.% MikroCaps caused a 6% decrease in apparent density, and this was the largest change. The 15 wt.% GR42 additive reduced apparent density by 4%, and 15 wt.% PX25 reduced apparent density by only 1%. The decrease in apparent density could have been due to inaccurate mixing of the ingredients during sample manufacture or failure of the ingredients to all react;
- The highest porosity in the large region was found in the sample with 15 wt.% MikroCaps—51%, while in the small closed region, the highest porosity was observed in the sample with 15 wt.% GR42—81%. The more meaningful result was from the small closed region, so the higher porosity was obtained by the sample with 15 wt.% GR42;
- Fly ash is a type of inert waste. When alkaline solutions are added to the geopolymer mixture, the produced product should be treated as hazardous waste. All tested samples had lower permissible values of all elements listed in the table (for hazardous waste). Regarding the pH of the tested samples, each of them showed an alkaline pH, due to the activation of the polycondensation process with an alkaline solution of NaOH;
- The MikroCaps phase-change additive did not affect the compressive strength. The GR42 additive increased the compressive strength by 30%, while PX25 decreased this parameter by 43%;
- The thermal conductivity of all samples oscillated at the same level of 0.08 W/m·K. The thermal conductivity for the 15 wt.% MikroCaps sample decreased by 0.1%, for the 15 wt.% GR42 sample, it decreased by 4%, while for the 15 wt.% PX25 sample, it increased by 9%. All the thermal conductivity values were very low, even though the thickness of these samples was as much as 10 cm;
- The volumetric heat capacity for each sample with PCM increased significantly, a very welcome effect. The largest increase was observed for the sample with 15 wt.% MikroCaps—a 41% increase. The Cv for the sample with 15 wt.% GR42 increased by 13%, and for the sample with 15 wt.% PX25, it increased by 41%;
- All samples with PCM had a higher Cp value than the reference sample. The Cp of the sample with 15 wt.% MikroCaps increased by 45%, that of the sample with 15 wt.% GR42 increased by 19%, and that of the sample with 15 wt.% PX25 increased by 44%;
- The coefficient of should be as low as possible because then there is greater susceptibility of the material to temperature equilibration. All samples with PCMs had a lower ratio than the reference sample. For the sample with 15 wt.% MikroCaps, the thermal diffusivity decreased by 23%, for the sample with 15 wt.% GR42 by 14%, and for the sample with 15 wt.% PX25, it decreased by 20%;
- Very favorable results were obtained for all parameters that should improve with the addition of PCMs, so all tests confirmed the performance effect of phase-change materials in geopolymer foams. All samples obtained the same confidence level—95%, meaning that 95% of the measurement results were within the real range.

Based on the results of this study, it can be concluded that the particle size of the phase-change materials used had a significant effect on the porosity. The average particle size of the MikroCaps additive resulted in the formation of fine and homogeneous pores. With a slightly larger particle size than MikroCaps, PX25 obtained medium-sized homogeneous pores. The large particle size of the GR42 additive made the pores quite heterogeneous and of different sizes.

Based on the above test results, it can be concluded that the best parameters were obtained for the sample with 15 wt.% MikroCaps. For this sample, all thermal parameters that could have improved did so to the greatest extent. Samples with PCMs should have low thermal diffusivity and high specific heat and heat capacity, all of which were achieved to the greatest extent for this sample. The other samples also achieved satisfactory results, and in each case, the positive effect of the phase-change additives for the geopolymer foams was confirmed. Geopolymer foams with PCM additives are a topic of great interest and commercial potential, and given the growing demand for new environmentally friendly and energy-efficient solutions for building insulation, the topic will persist. Geopolymer foams are very good substitutes for commercial products such as polycyclic isocyanates and wools.

## Figures and Tables

**Table 1:** Oxide analysis for fly ash.

Precursor	Oxide Composition (wt.%)					
	SiO_2_	Al_2_O_3_	K_2_O	FeO	Na_2_O	MgO
**Fly ash**	55.82	34.48	4.55	1.88	1.81	1.46

**Table 2:** Mineral phase analysis for fly ash.

Fly Ash			
Identified Phase	Chemical Formula	Percentage Share [wt.%]	Datasheet Number
Mullite	Al_6_Si_2_O_13_	51.3	00-015-0776
Quartz	SiO_2_	44.5	01-074-1811
Anhydrite	CaSO_4_	2.3	00-006-0226
Hematite	Fe_2_O_3_	0.7	04-006-2616
Magnetite	Fe_3_O_4_	0.7	04-006-6550
Rutile	TiO_2_	0.5	00-034-0180

**Table 3:** Technical parameters of PCMs used in geopolymer foams [41,42].

	MikroCaps	GR42	PX25
Melting point [°C]	28	38–43	22–25
Solidification temperature [°C]	-	43–37	25–22
Heat capacity [kJ/kg]	174	55	95
Specific heat [kJ/kg·K]	2	2	2
Thermal conductivity [W/m·K]	PCM alone: 0.2, in dry capsules: 0.4	0.2	0.1
PCM content [%]	35.6	30	60
Appearance	white suspension 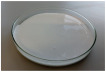	brown granules 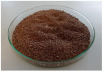	white powder 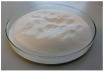

**Table 4:** Particle size analysis of PCMs.

Material	D_10_ [μm]	D_50_ [μm]	D_90_ [μm]	Average Value [μm]	Standard Deviation [μm]
MikroCaps	2.22	5.79	9.36	6.20	0.003
GR42	46.36	109.93	145.08	108.65	0.267
PX25	2.96	10.56	23.51	12.74	0.096

**Table 5:** Composition of ready-made samples with PCMs.

ID	Fly Ash [wt.%]	Cement [wt.%]	Sand [wt.%]	Microsphere [wt.%]	Surfactant [wt.%]	PCM Content [wt.%]	H_2_O_2_ [wt.%]	10 M Solution [wt.%]
R.F.A.	70	9	7	14	0.005	0	0.05	0.35
15% MikroCaps	70	9	7	14	0.005	15	0.05	0.35
15% GR42	70	9	7	14	0.005	15	0.05	0.40
15% PX25	70	9	7	14	0.005	15	0.05	0.52

**Table 6:** Calculated values of apparent density ρ_b1_ of the tested samples.

Designation of Samples	R.F.A.	15% MikroCaps	15% GR42	15% PX25
ρ_b1_ [kg/m^3^]	422.50	399.00	406.00	419.50

**Table 7:** Porosity of samples with PCM addition.

ID	Porosity in Large Region/ Visualization of Pores [%]	Porosity in Small Closed Region [%]
15% MikroCaps	51	66
15% GR42	48	81

**Table 8:** The results of testing the concentrations of harmful substances with the limit values for all samples.

Permissible Leaching Limits *				R.F.A.	15% MikroCaps	15% GR42	15% PX25
Liquid/Solid Phase = 10 L/kg [mg/kg Dry Weight] Baseline Test				mg/kg	mg/kg	mg/kg	mg/kg
Component	Criteria for Allowing Inert Waste to Be Deposited in an Inert Waste Landfill	Criteria for Allowing Hazardous Waste to Be Disposed of in a Hazardous Waste Landfill	Criteria for Allowing Non-Hazardous and Inert Waste to Be Deposited in a Landfill for Non-Hazardous and Inert Waste				
Arsen (As)	0.5	25	2	7.0	5.1	5.2	6.5
Bar (Ba)	20	300	100	0.20	0.23	0.22	0.21
Cadmium (Cd)	0.04	5	1	<0.0020	<0.0020	<0.0020	0.0038
Total chromium (Cr)	0.5	70	10	0.28	0.21	0.31	0.28
Copper (Cu)	2	100	50	0.28	0.33	0.22	0.28
Mercury (Hg)	0.01	2	0.2	<0.010	<0.010	<0.010	<0.010
Molybdenum (Mo)	0.5	30	10	3.5	1.7	1.6	2.1
Nickel (Ni)	0.4	40	10	0.051	0.087	0.054	0.062
Lead (Pb)	0.5	50	10	0.028	<0.020	0.043	0.038
Antimony (Sb)	0.06	5	0.7	0.24	0.15	<0.020	0.25
Selen (Se)	0.1	7	0.5	1.7	1.3	1.3	1.2
Zinc (Zn)	4	200	50	0.23	0.17	0.20	0.24
Chlorides (Cl^−^)	800	25,000	15,000	250	230	250	190
Fluorides (F^−^)	10	500	150	40	36	36	51
Sulfates (SO_4_^2−^)	1000	50,000	20,000	4100	4200	3500	3800
Dissolved organic carbon (DOC)	500	1000	800	2700	3300	3000	1200
Dissolved solids (TDS) **	4000	100,000	60,000	48,000	53,000	45,000	62,000
Chromium ^6 +^				38	41	48	55
pH				11.5	11.1	11.6	10.8

* Permissible leaching limits for waste disposed in landfills equipped with leachate collection systems subsequently directed to wastewater treatment plants, except for DOC and TDS components, which are considered to be met for values higher than those specified in the table. ** Values for dissolved solid compounds (TDSs) can be used interchangeably for sulfate and chloride values.

**Table 9:** Compressive strength for all samples.

ID	R_c_ [MPa]
R.F.A.	0.7
15% MikroCaps	0.7
15% GR42	0.9
15% PX25	0.4

**Table 10:** Average values of thermal parameters for all samples.

ID	Thermal Properties of Samples with PCMs			
	λ [W/(m × K)]	C_v_ [MJ/(m^3^ × K)]	C_p_ [J/(kg × K)]	α [mm^2^/s]
R.F.A.	0.0839	0.4900	1182.7	0.1703
15% MikroCaps	0.0838	0.6914	1717.6	0.1318
15% GR42	0.0802	0.5544	1402.3	0.1462
15% PX25	0.0911	0.6888	1705.8	0.1358

**Table 11:** Calculated confidence intervals of measured thermal properties of modified geopolymer materials.

Test Sample	Determined Confidence Intervals
R.F.A.	P (0.0837 ≤ λ ≤ 0.0840) = 0.95
	P (1178.6 ≤ C_p_ ≤ 1186.5) = 0.95
	P (0.1697 ≤ α ≤ 0.1708) = 0.95
15% MikroCaps	P (0.0741 ≤ λ ≤ 0.0933) = 0.95
	P (1647.5 ≤ C_p_ ≤ 1787.6) = 0.95
	P (0.1214 ≤ α ≤ 0.1421) = 0.95
15% GR42	P (0.07947 ≤ λ ≤ 0.0809) = 0.95
	P (1396.3 ≤ C_p_ ≤ 1408.3) = 0.95
	P (0.1455 ≤ α ≤ 0.1468) = 0.95
15% PX25	P (0.0900 ≤ λ ≤ 0.0922) = 0.95
	P (1682.5 ≤ C_p_ ≤ 1729.2) = 0.95
	P (0.1328 ≤ α ≤ 0.1388) = 0.95

## Data Availability

Data are contained within the article.
